# Effects of Resveratrol on Intestinal Flora and Metabolism in Rats With Non‐Steroidal Anti‐Inflammatory Drug‐Induced Intestinal Injury Under Plateau Hypoxia Environment

**DOI:** 10.1002/fsn3.70228

**Published:** 2025-05-20

**Authors:** ShengLong Xue, Tian Shi, Weidong Liu, Yan Feng, Ailifeire Tuerxuntayi, Na Li, Feng Gao

**Affiliations:** ^1^ College of Life Science and Technology Xinjiang University Urumqi China; ^2^ Department of Gastroenterology People's Hospital of Xinjiang Uygur Autonomous Region Urumqi China; ^3^ Xinjiang Clinical Research Center for Digestive Diseases Urumqi China; ^4^ Xinjiang Medical University Urumqi China

**Keywords:** 16SrRNA sequencing, metabolites, non‐steroidal anti‐inflammatory drugs, plateau hypoxic environment, resveratrol

## Abstract

The plateau hypoxic environment is a main habitat for human beings, which can result in dysbiosis of the intestinal flora. Non‐steroidal anti‐inflammatory drugs (NSAIDs) are widely used anti‐inflammatory drugs that can cause intestinal damage with long‐term administration. Moreover, the administration of these drugs in the hypoxic plateau environment may exacerbate intestinal damage. This study aimed to investigate the therapeutic effect of resveratrol (RSV) on the intestinal injury induced by NSAIDs in rats under plateau hypoxia. Aspirin was used as the inducer to induce intestinal injury in rats. Rats were divided into seven groups: Ck (vehicle group), HCk (high‐altitude control group), PAsp (plain aspirin‐treated group), HAsp (High‐altitude aspirin‐treated group), RSVL (low‐dose resveratrol‐treated group), RSVM (medium‐dose resveratrol‐treated group), and RSVH (high‐dose resveratrol‐treated group). The body weight of the rats was recorded every 7 days during the experiment. On the last day of the experiment, jejunal tissues of the rats were collected for hematoxylin and eosin staining (H&E), and feces of the rats were collected for analysis of intestinal flora and metabolite analysis. The results demonstrated that RSV inhibited weight loss and intestinal damage initiated by aspirin administration in a hypoxic plateau environment. Moreover, it markedly elevated the expression levels of interleukin (IL)‐10 and superoxide dismutase (SOD) while substantially reducing the expression levels of TNF‐α, IL‐1β, and myeloperoxidase (MPO). Furthermore, 16SrRNA gene sequence analysis showed that both aspirin and RSV altered the composition and structure of rat gut microbiomes. Metabolomics results showed that RSV altered the intestinal metabolites of aspirin‐induced intestinal injury in rats, reducing the content of 2‐hydroxy‐3‐ (4‐hydroxyphenyl) protonic acid and 3‐ [(1‐carboxyvinyl) oxy] benzoate and increasing the content of coumaryl, 3‐amino‐4 hydroxybenzoate, and L‐carnitine. Resveratrol can alleviate NSAID (Aspirin)‐induced intestinal damage in the hypoxic environment of the plateau by regulating intestinal flora and metabolites, with the best effect in the RSVM group (50 mg/kg).

## Graphical abstract

In the present study, we investigated the mitigating effect of resveratrol on intestinal injury induced by NSAIDs in a plateau hypoxia model by detecting cytokines (IL‐10, TNF‐α, and IL‐1β), oxidative stress markers (SOD and MPO), 16S rRNA, and serum metabolomics. We found that resveratrol could alleviate intestinal inflammation and ameliorate intestinal injury by modulating the expression of inflammatory factors and oxidative stress markers SOD and MPO. Furthermore, resveratrol maintains intestinal health by regulating intestinal microorganisms and serum metabolites.

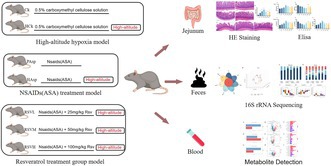

## 1 Background

The plateau environment is one of the main environments for human survival, characterized by low pressure, hypoxia, and strong ultraviolet rays (Han et al. 2024; Zhong et al. 2024; Richalet, Jeny, et al. 2023). In the hypoxic environment of high altitude, severe threats exist to human health, especially damage to the gut and disruption of the gut microbiota (Liu et al. 2024). This environment results in elevated levels of oxidative stress and inflammation, which in turn disrupt gut barrier function, giving rise to increased intestinal permeability, bacterial translocation, and local and systemic inflammatory responses (Ma et al. 2023). Moreover, prolonged exposure to high‐altitude hypoxia may enhance the risk of cognitive decline potentially due in part to hypoxia‐induced alterations in gut microbial composition (Zhou, Zhou, et al. 2024). Multiple studies have demonstrated that exposure to high‐altitude hypoxia severely disrupts the gut microecology, inflicting damage on the intestinal tract and intestinal mucosal barrier and modifying the composition of the gut microbiota (Yu et al. 2023; Shah 2016; Wang, Liu, et al. 2023). The modification of the intestinal microbiota under high‐altitude hypoxia may be one of the contributing factors to the frequent incidence of various intestinal diseases in high‐altitude populations (Lewis et al. 2023).

Non‐steroidal anti‐inflammatory drugs (NSAIDs), such as aspirin, ibuprofen, and meloxicam, are a class of anti‐inflammatory drugs devoid of glucocorticoids, widely utilized for their anti‐inflammatory, anti‐rheumatic, analgesic, and anticoagulant properties, effectively alleviating pain and inflammation (Wirth et al. 2023). However, NSAIDs are associated with multiple side effects, such as damage to the mucosa of the gastrointestinal tract and disruption of intestinal flora homeostasis, resulting in flora imbalance (Pratama et al. 2024; Simon O'Brien et al. 2023; Queiro‐Silva et al. 2021). Multiple studies have demonstrated that long‐term use of NSAIDs can induce intestinal damage, disrupt the intestinal mucosal barrier, trigger intestinal inflammation, and contribute to the development of a variety of intestinal diseases (Wang, Shi, et al. 2023; Chang et al. 2011; Watanabe et al. 2020; Feng et al. 2024). Administration of NSAIDs in populations in high‐altitude hypoxic areas may further intensify the degree of intestinal damage (Xue et al. 2024). Although long‐term NSAIDs‐induced intestinal injury has received extensive attention, no studies have been carried out to explore the effects of NSAIDs on intestinal injury and intestinal flora in high‐altitude hypoxic environments.

In the context of the combined stress of the plateau environment and non‐steroidal anti‐inflammatory drugs (NSAIDs), the use of NSAIDs in the plateau hypoxic environment may further exacerbate intestinal damage and intestinal flora disruption. Currently, research on relevant interventions and treatments is extremely limited. Resveratrol (RSV) is a polyphenolic organic compound with a diverse range of pharmacological effects, including antioxidant and anti‐inflammatory properties. It is mainly extracted from plants such as tiger balm, cranberries, thuja, grapes, and so on. (Gostimirovic et al. 2023; Radwan and Karam 2020). RSV, as a molecule of compounds extracted from natural products, has been well studied, and due to its multifaceted properties, RSV has therapeutic effects (Zhang and Kiarasi 2024; Ghaeini Hesarooeyeh et al. 2024; Bi et al. 2023). RSV exhibits a protective effect on the intestinal tract and may function in protecting the intestinal tract by modulating the intestinal flora (Gostimirovic et al. 2023). Studies have demonstrated that RSV can alleviate hepatic fibrosis via the microbial‐intestinal‐hepatic axis by modulating the intestinal flora, indicating that RSV can indirectly influence the function and health status of distal organs by modulating the intestinal flora (Li et al. 2024). Moreover, studies have indicated that RSV can modify the gut microbial structure and mitigate intestinal injury, thereby enhancing intestinal health (Zhou, Zeng, et al. 2024). A systematic review by Sandoval‐Ramírez et al. (2021) described the effects of phenolic compounds on the intestinal barrier. As a typical phenolic compound, RSV was found to be effective in animal studies in improving the integrity of the intestinal barrier and reducing intestinal injury. Therefore, RSV can be used as a good prophylactic drug in inflammation prevention and protection of the intestinal barrier.

The purpose of this study was to simulate the plateau hypoxic environment, administer aspirin via gavage to rats within this environment, and investigate the impact of NSAID administration on intestinal damage in rats under plateau hypoxic conditions. Through RSV intervention, we investigated its potential to ameliorate intestinal inflammation induced by NSAID administration in rats under the plateau hypoxic environment. We also examined its impact on intestinal flora and metabolites, aiming to offer valuable insights for preventing intestinal diseases resulting from NSAID administration under plateau hypoxic conditions.

## 2 Materials and Methods

### 2.1 Reagents and Drugs

Aspirin enteric‐coated Tablets (100 mg/Tablet) were purchased from Bayer Healthcare GmbH, Germany. Resveratrol was purchased from Sigma Company, USA, with 98% purity, commodity number: V900386. Pentobarbital sodium was purchased from Sinopharm Chemical Reagent Co. Ltd. Carboxymethyl cellulose sodium was purchased from Shanghai Macklin Reagent Co. Ltd. The ELISA assay kits were purchased from Merck.

### 2.2 Experimental Animals and Environment

This experiment was approved by the Animal Ethics Committee for Research Experiments of Xinjiang Medical University (Approval number: KY20230209135) and conducted in accordance with the ARRIVE guidelines. Seventy male SD rats of SPF grade were purchased from Xinjiang Medical University (Xinjiang, China) and housed in the Northwest Key Laboratory of Special Environmental Medicine of Xinjiang Military Region General Hospital of the Armed Police (Xinjiang, China). Plateau hypoxia environment in the laboratory developed ‘special environment artificial test chamber’, which was configured to simulate an environment of 5500 m altitude, 379 mmHg, with the temperature controlled at 20°C ± 2°C.

The initial body weights of all rats ranged from 160 to 180 g. The rats were kept at a room temperature of 20°C. They were pre‐housed for 1 week in an SPF‐grade animal laboratory at a room temperature of 20°C ± 2°C and a humidity of (45% ± 5%) RH, with free access to water and food to acclimatize to the environment. After 1 week of pre‐feeding, the rats were placed into a plateau hypoxia simulation chamber to simulate the hypoxic environment of the plateau. The test rats were kept in the plateau hypoxic environment except during the time of gavage (12:30–1:30 every day) (Xue et al. 2024).

### 2.3 Experimental Design and Weight

Some of the research methods and experimental designs used in this study are the same as those used in our team's previous research. Some of the results are from our previous research and have been cited in the corresponding sections of the article (Xue et al. 2024). In this study, seventy rats were randomly assigned into 7 groups (*n* = 10) and numbered according to the assigned groups. They were divided into seven 7 groups: the vehicle control group (100 m above sea level, 0.5% carboxymethyl cellulose solution gavage), the plain aspirin‐treated group (100 m above sea level, gavage with 200 mg/kg of aspirin), the high‐altitude control group (entering a simulated low‐pressure oxygen chamber, gavage with 0.5% carboxymethyl cellulose solution), and the high‐altitude aspirin‐treated group (entering a simulated low‐pressure oxygen chamber, gavage with 200 mg/kg of aspirin). The RSV‐treated group (entering a simulated low‐pressure oxygen chamber, gavage with 200 mg/kg aspirin, followed by resveratrol 30 min later)includes the low‐dose resveratrol‐treated group (25 mg/kg resveratrol), medium‐dose resveratrol‐treated group (50 mg/kg resveratrol), and high‐dose resveratrol‐treated group (100 mg/kg resveratrol) (Xue et al. 2024; Sandoval‐Ramírez et al. 2021; Zhao et al. 2023).

Preparation of resveratrol solution is given as follows: Dissolve 5 g of carboxymethyl cellulose sodium (CMC‐Na) in 1000 mL of deionized water to form a 0.5% carboxymethyl cellulose solution and dissolve resveratrol powder in the carboxymethyl cellulose solution to form a 50 mg/mL resveratrol solution. Aspirin was configured in a similar manner (Liang et al. 2022). The aforementioned gavage treatment regimen was continued for 3 weeks. After the last gavage, the rats were given water but deprived of rat food, then weighed 12 h later, and subsequently anesthetized with 2% pentobarbital sodium intraperitoneally; blood samples were collected from the retro‐orbital plexus, and then, the animals were euthanized using cervical dislocation. All rats were dissected, and intestinal jejunal tissue was dissected out, rinsed with saline to eliminate blood contamination, and then preserved in PBS (phosphate buffered saline) solution for various subsequent biochemical and histopathological examinations. Feces from the intestines of rats were collected and retained for subsequent microbiological analysis.

### 2.4 Histological and Biochemical Analysis

The jejunal specimens were immersed in a 10% neutral formaldehyde solution for fixation, routinely dehydrated, and then subjected to paraffin‐embedded sectioning to prepare 5‐μm‐thick sections for morphological evaluation. Subsequently, the sections were deparaffinized using xylene, sequentially washed with a series of graded alcohols, and then stained with hematoxylin and eosin (H&E). Finally, the intestinal mucosal changes were observed under a light microscope, and the intestinal damage was scored according to Chiu's scale (Chiu et al. 1970; Wang, Shen, et al. 2023) (Table S1).

### 2.5 16sRNA Sequencing for Gut Microbial Analysis

Six rats were randomly selected from each group, and their feces were collected for intestinal microbial analysis. The DNA samples were extracted from the rat fecal samples using the TopTaq DNA polymerase kit (Transgen, China). Subsequently, the DNA samples were quantified using a NanoDrop 2000 spectrophotometer (Thermo Scientific, Wilmington, NC, USA) and assessed on a 1% agarose gel. The specific primer pairs with barcodes (forward primer 5′‐CCTACGGGGNGGCWGCAG‐3′ and reverse primer 5′‐GACTACHVGGGTATCTAATCC‐3′) were used to amplify the V3‐V4 highly variable region of the 16S rRNA gene. Each sample was independently amplified three times. The PCR products were then analyzed by agarose gel electrophoresis and combined from the same sample. The combined PCR products were used as templates for index PCR, incorporating index primers to add the Illumina index to the library. The amplification products were visualized via gel electrophoresis and purified using the Agencourt AMPure XP Kit (Beckman Coulter, CA, USA). The purified products were cataloged into libraries targeting the 16S V3‐V4 region. The quality of the libraries was assessed using the Qubit@2.0 Fluorometer (Thermo Scientific) and the Agilent Bioanalyzer 2100 system. Lastly, the combined libraries were sequenced using an Illumina MiSeq 250 sequencer, producing paired‐end reads of 2 × 250 bp.

### 2.6 Fecal Metabolome Analysis

Fecal samples were accurately weighed in 2 mL EP tubes, and then, 600 μL of methanol (containing 2‐chloro‐L‐phenylalanine (4 ppm)) was added. The samples were incubated at −20°C and vortexed for 30 s. Steel beads were added to the tubes and then put into a tissue grinder and ground at 50 Hz for 120 s. The ground samples were sonicated for 10 min at room temperature and then centrifuged at 12,000 rpm at 4°C for 10 min. The upper supernatant was filtered through a 0.22 μm membrane and added to the assay bottle for detection. The liquid chromatography‐mass spectrometry (LC/MS) system used for metabolomics analysis comprised a Thermo Vanquish ultra‐high performance liquid chromatography (UPLC) system and a Thermo Orbitrap Exploris 120 mass detector. Details of the electrospray ionization (ESI) parameters are provided in the Supporting Information.

### 2.7 Data Statistics and Analysis

The results are presented as means ± standard deviation (SD). One‐way analysis of variance and Duncan's post hoc test were used to test statistical significance (SPSS 26 software, IBM, Chicago, IL, USA). Graphing was done through Graphpad prism 8.0 software. Univariate and bivariate correlation analysis was performed through RStudio. *p* < 0.05 indicates a significant difference. The * in this study represents comparisons with the plains blank group: *p < 0.05, **p < 0.01, and ***p < 0.001, and # represents the comparisons with the plateau NSAID‐treated group: *#p* < 0.05, *##p* < 0.01, *###p* < 0.001.

## 3 Results

### 3.1 Weight Changes in Rats

**FIGURE 1:**
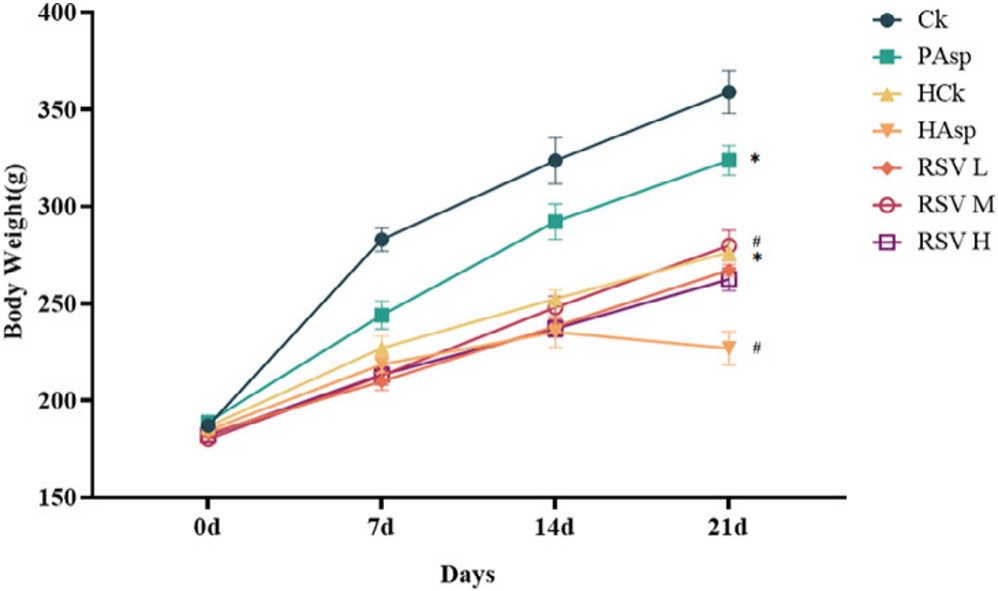
Changes in body weight of rats in different treatment groups. (Ck) Vehicle group; (PAsp) plain aspirin‐treated group; (HCk) high‐altitude control group; (HAsp) high‐altitude aspirin‐treated group; (RSVL) 25 mg/kg resveratrol‐treated group; (RSVM) 50 mg/kg resveratrol‐treated group; and 100 mg/kg high‐dose resveratrol‐treated group (RSVH). hCk, PAsp, and HAsp compared with Ck group, *p < 0.05; RSVL, RSVM, and RSVH compared with HAsp group, #*p* < 0.05.

Compared to the Ck group, the PAsp, HCk, and HAsp groups all exhibited significantly diminished weight gain, with the HAsp group displaying the slowest rate (*p* < 0.05). Rats in the HAsp group commenced losing weight starting from the 14th day of the study. Following intervention with RSV, no significant changes were observed in the body weight of rats on the 7th and 14th days compared to the HAsp group. However, starting from the 14th day, rats treated with RSV exhibited higher body weight gain than those in the HAsp group. Moreover, the effect of body weight gain was more pronounced in the RSVM group compared to the RSVL and RSVH groups (Table S1) (*p* < 0.05) (Figure 1).

### 3.2 HE Staining of Rat Jejunum

**FIGURE 2:**
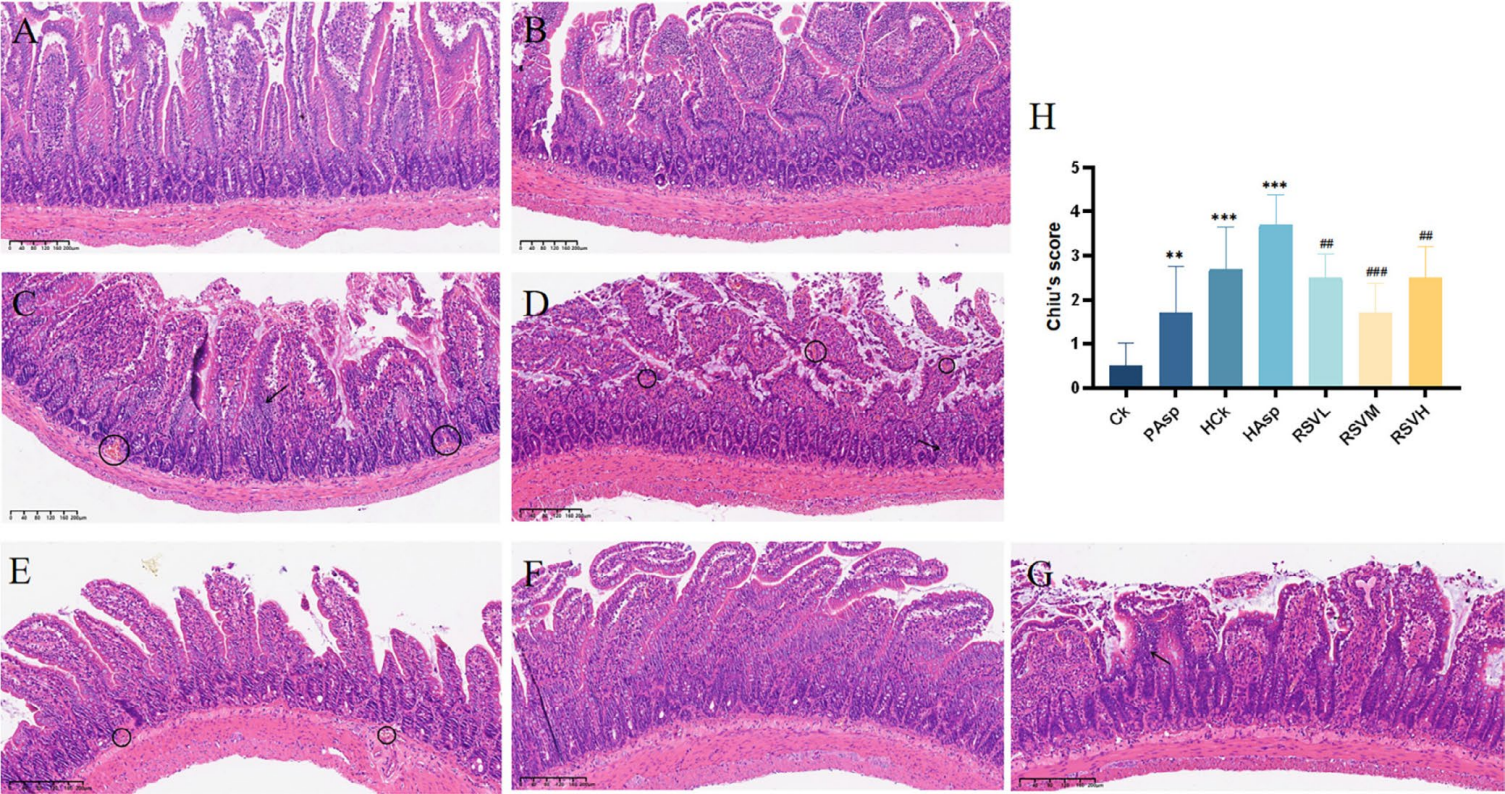
Histopathological section results of rat jejunum. (A) Vehicle group (Ck); (B) plain Aspirin‐treated group (PAsp);(C) high‐altitude control group (HCk); (D) high‐altitude aspirin‐treated group (HAsp); (E) low—dose resveratrol—treated group (RSVL); (F) medium—dose resveratrol—treated group (RSVM); (G) high—dose resveratrol—treated group (RSVH); (H) Chiu's of the jejunal tissue score. Red arrow: bleeding points, black arrow: inflammatory cell infiltration, and blue arrow: villus atrophy or shedding.

The histopathological results of the jejunum in different treatment groups were observed through HE staining, as depicted in Figure 1. The small intestinal mucosal villi appeared normal in the Ck group, with tightly arranged cells and no apparent pathological changes (Figure 2A). Intestinal mucosal villi in the PAsp group exhibited atrophy; the lamina propria was edematous, and a few epithelial cells were necrotic (Figure 2B). Intestinal mucosal villi in the HCk group were atrophied, with partial detachment of the villi, and the pathology section revealed multiple hemorrhagic spots and infiltration of inflammatory cells (Figure 2C). In the HAsp group, the intestinal mucosal villi were severely atrophied, with extensive areas of detachment, and the infiltration of inflammatory cells was pronounced, accompanied by multiple hemorrhagic spots (Figure 2D). Following RSV intervention, the intestinal damage was significantly reduced, with minimal shedding of intestinal villi, a notable decrease in the number of hemorrhagic dots, and the resolution of lamina propria edema (Figure 2E–G). These findings demonstrate the effective reduction of intestinal damage caused by aspirin administration under plateau hypoxia through RSV intervention, with the RSVM group exhibiting the most significant effect (Xue et al. 2024).

### 3.3 Effect of RSV on Jejunal Inflammatory Cytokines as Well as Blood Enzyme Activities in Rats With Aspirin‐Induced Intestinal Injury

**FIGURE 3:**
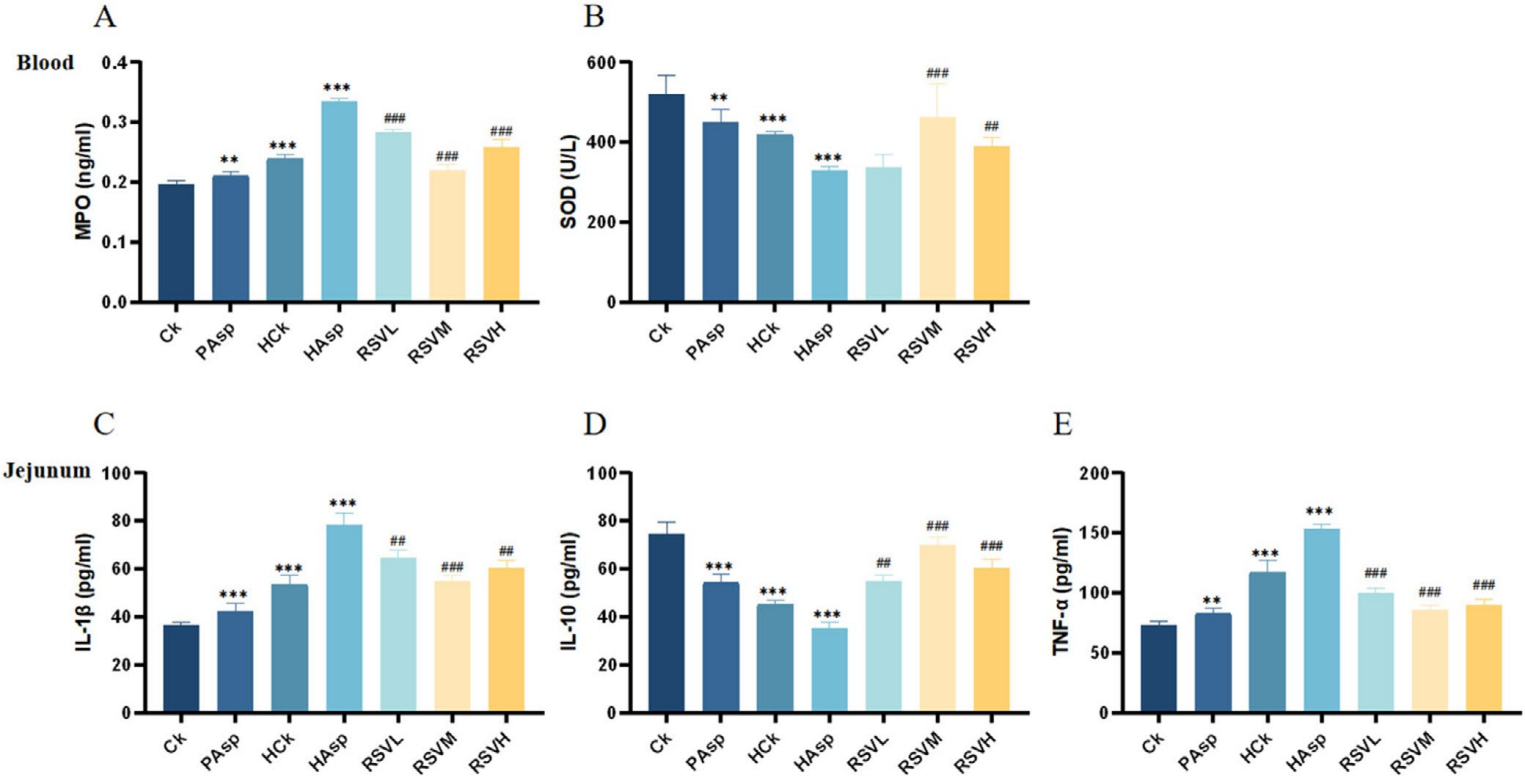
(A, B) Blood oxidative stress indices. (A) Changes of MPO in different treatment groups; (B) Changes of SOD in different treatment groups. (C–E) Expression of jejunal inflammatory factors. (C) jejunal IL‐1β; (D) jejunal IL‐10; (E) jejunal TNF‐α.

Oxidative stress indicators are believed to contribute to the onset and perpetuation of intestinal inflammation (Jiang et al. 2023). We assessed the expression of oxidative stress indicators, namely, myeloperoxidase (MPO) and superoxide dismutase (SOD), in the blood serum using the ELISA method (Figure 3A,B). MPO and SOD were expressed at varying levels among different treatment groups. We found that a plateau hypoxic environment stimulates an increase in MPO production and a decrease in SOD production in rats. In addition, aspirin administration under plateau conditions further increased the level of MPO while decreasing the level of SOD. After treatment with RSV, compared with the HAsp group, MPO levels decreased in all three RSV–intervention groups, with the most significant decrease in the RSVM group (*p* < 0.05). SOD levels exhibited an upward–regulation trend, with the most significant upward–regulation in the RSVM group (*p* < 0.05).

The study examined the levels of pro‐inflammatory cytokines interleukin 1‐β (IL‐1β), interleukin‐10 (IL‐10), and tumor necrosis factor‐α (TNF‐α) in the serum and jejunum of rats. Results showed that expression levels of IL‐1β and TNF‐α were significantly higher in rats exposed to plateau hypoxic conditions than in the control group (Ck), with lower levels of IL‐10 observed. These findings suggest that plateau hypoxic conditions induce the release of pro‐inflammatory cytokines and cause intestinal inflammation. Moreover, aspirin administration under plateau conditions increased IL‐1β and TNF‐α release while decreasing IL‐10 release. The jejunal expression levels of IL‐1β and TNF‐α were higher in the aspirin‐treated group than in the control group (HCk), with lower levels of IL‐10 being observed in the former. RSV intervention significantly reduced the production of IL‐1β and TNF‐α in jejunal tissues and increased IL‐10 expression levels (Figure 3C–E). The RSVM group (50 mg/kg) was found to be the most effective at reducing intestinal damage caused by NSAID administration under plateau hypoxic conditions (Xue et al. 2024).

### 3.4 Effect of RSV on Gut Microorganisms in Rats With Aspirin‐Induced Intestinal Injury

**FIGURE 4:**
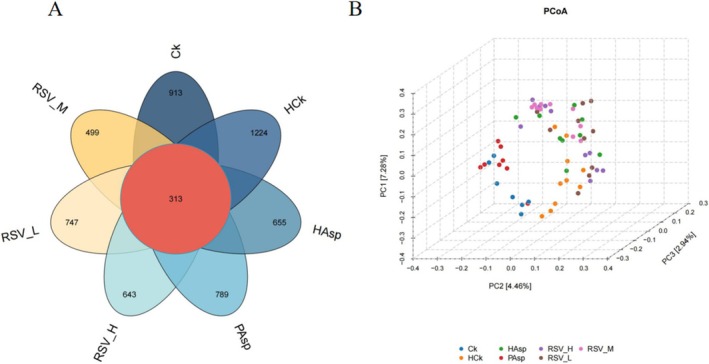
Effects of different treatment groups on β‐diversity in rats. (A) Venn diagram. (B) PCoA‐3D plot.

In order to investigate the effects of plateau hypoxic environments and aspirin on the intestinal flora of rats and to determine whether RSV can regulate the diversity of intestinal microorganisms in rats with aspirin‐induced intestinal injury, we collected rat feces for high‐throughput sequencing analysis of the 16S rRNA gene. The uniqueness and similarity of different samples were represented by a Venn diagram (Figure 4A). The number of OTUs in the Ck, PAsp, HCk, HAsp, RSVL, RSVM, and RSVH groups was 1226, 1102, 1537, 968, 1060, 812, and 956, respectively. The number of OTUs shared by the seven groups was 313, indicating that the species and number of microorganisms differed among the seven groups. In addition, we further analyzed the α and β diversity. There was no significant difference in the Shannon index between the HCk and PAsp groups when compared to the Ck group (Figure S1). The Shannon index showed a significant decrease in the HAsp group compared to the HCk group (*p* < 0.05) and exhibited a significant decrease in the HAsp group compared to the PAsp group (*p* < 0.05). The Shannon index did not show a significant difference in the RSVL, RSVH, and RSVM groups when compared to the HAsp group, suggesting a notable alteration in gut microbial abundance by aspirin in rats under plateau conditions. In contrast, RSV had no impact on the modification of gut microbial abundance. Analyses of the ACE index and Simpson's index demonstrated that both aspirin and RSV treatments did not affect species diversity (Figure S1). This outcome implies that aspirin and RSV possess constraints in modulating species richness and diversity. The comparison of gut microbial diversity among the seven groups was conducted using β‐diversity. The results of principal coordinate analysis (PCoA) revealed variations in the intestinal flora among the seven groups of rats (Figure 4B).

**FIGURE 5:**
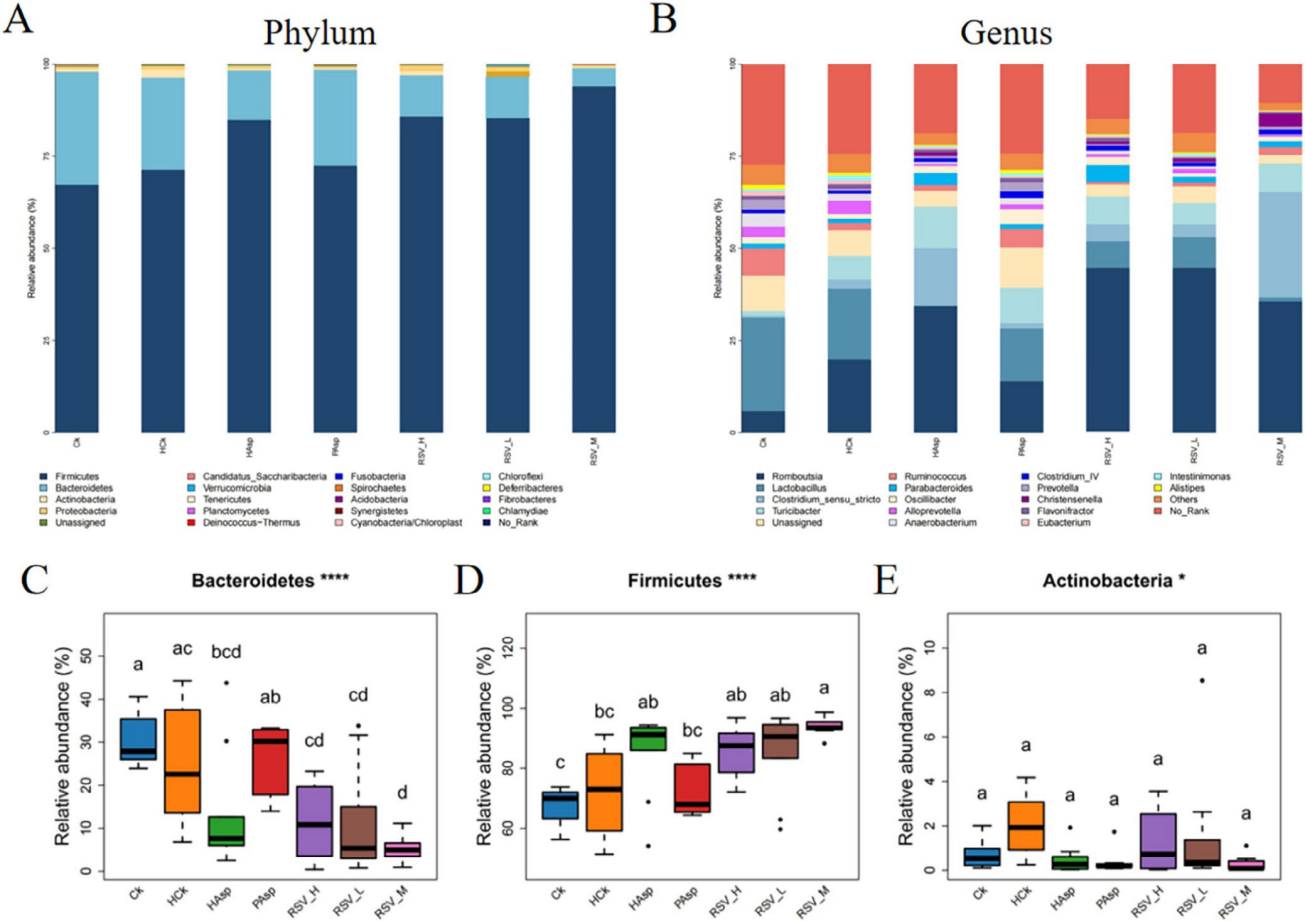
Changes in the abundance of rat intestinal flora in different treatment groups. (A) Relative abundance of flora at the phylum level in different treatment groups. (B) Relative abundance of flora at the genus level in different treatment groups. (C) Anaplasma phylum. (D) Thick‐walled phylum. (E) Actinobacteria phylum.

In addition, we investigated the impact of various treatment groups on microbial abundance across different taxonomic levels. The primary phylum‐level groups comprised *Firmicutes*, *Bacteroidetes*, and *Actinobacteria*, collectively constituting over 98% of the total floral population (Figure 5A). The relative abundance of *Firmicutes* varied across groups: 67.24% (Ck), 71.32% (HCk), 72.38% (PAsp), 84.86% (HAsp), 85.46% (RSVL), 93.97% (RSVM), and 85.82% (RSVH). These findings suggest a significant increase in *Firmicutes* abundance due to aspirin treatment. A total of 35 distinct genera exhibited differential abundance at the genus level (Figure 5B). Six bacterial taxa exhibited differential abundance between the Ck and HCk groups: *Ruminococcus*, *Facklamia*, *Parasutterella*, ascending *Jeotgalicoccus*, *Coprococcus*, and *Psychrobacter*, indicating that plateau hypoxic conditions influence the composition of rat intestinal flora at the genus level. Five bacterial taxa exhibited differences at the genus level between the HCk and HAsp groups: *Facklamia*, *Jeotgalicoccus*, *Roseburia*, *Psychrobacter*, and *Alloprevotella*. This suggests a similar effect of Aspirin gavage. Two bacterial taxa exhibited differential abundance between the RSVM and HAsp groups: *Clostridium_sensu_stricto* (up) and *Spirosoma* (up, *p* < 0.05). *Cupriavidus* was the sole bacterium exhibiting significant differential abundance between the RSVH and HAsp groups (up, *p* < 0.05). No significant differences were observed in bacterial taxa at the genus level between the RSVL and HAsp groups.

To comprehend the distinctions among groups, we identified microorganisms exhibiting significant differences between groups utilizing linear discriminant analysis (LDA) effect size (LEfSe). As depicted in Figure S2, the predominant microbial taxa in the Ck group comprised *Bacteroidetes*, *Ruminococcus*, and *Prevotellaceae*; in the HCk group, they included Alloprevotella, Intestinmonas, and *Roseburia*; the HAsp group exhibited dominance by *Ruminococcus2*, *Bardyrhizobiaceae*, and *Clostridiales_Incertae_sedis_XI*; the PAsp group displayed prevalence of *Ruminococcus_callidus*; the RSVH group featured *Peptostreptococcaceae* and *Romboutsia*; *Bilophila* was dominant in the RSVL group; and *Clostridiales, Clostridia*, and *Clostridium_sensu_stricto* were predominant in the RSVM group. These findings suggest that RSV has the potential to alter the composition and structure of microbial communities (Xue et al. 2024).

### 3.5 Analysis of Intestinal Metabolites

**FIGURE 6:**
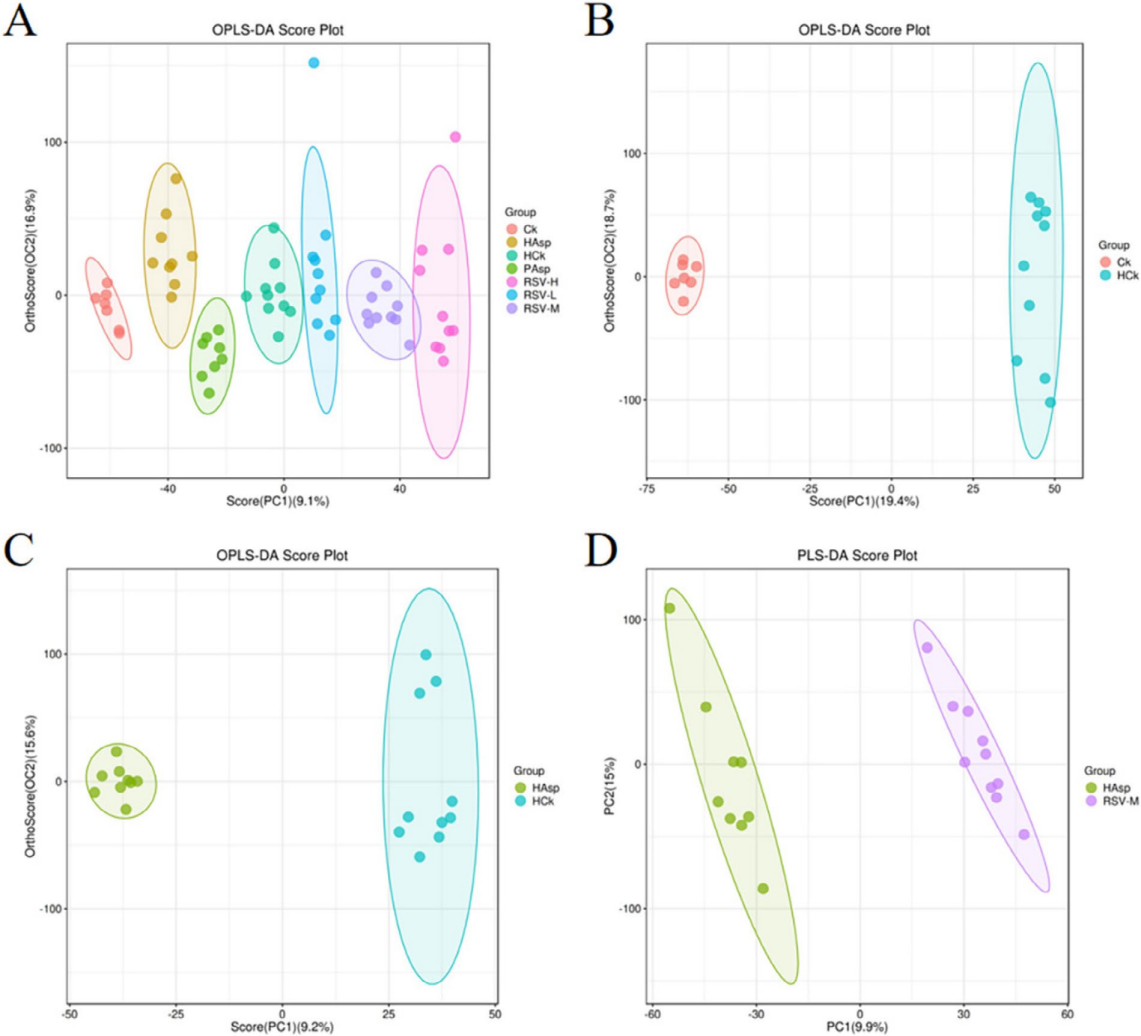
Multivariate statistical comparison plots between groups. (A) Plot of OPLS‐DA scores in different treatment groups. (B) HCk‐Ck. (C) HAsp‐HCk. (D) RSVM‐HAsp.

Metabolomic profiles of rat serum samples were analyzed using orthogonal OPLS‐DA score plots to understand the differences between the groups (Figure 6). OPLS‐DA showed that there was a separation of metabolic profiles between the seven groups, suggesting that there were differences in serum metabolites between the seven groups.

**FIGURE 7:**
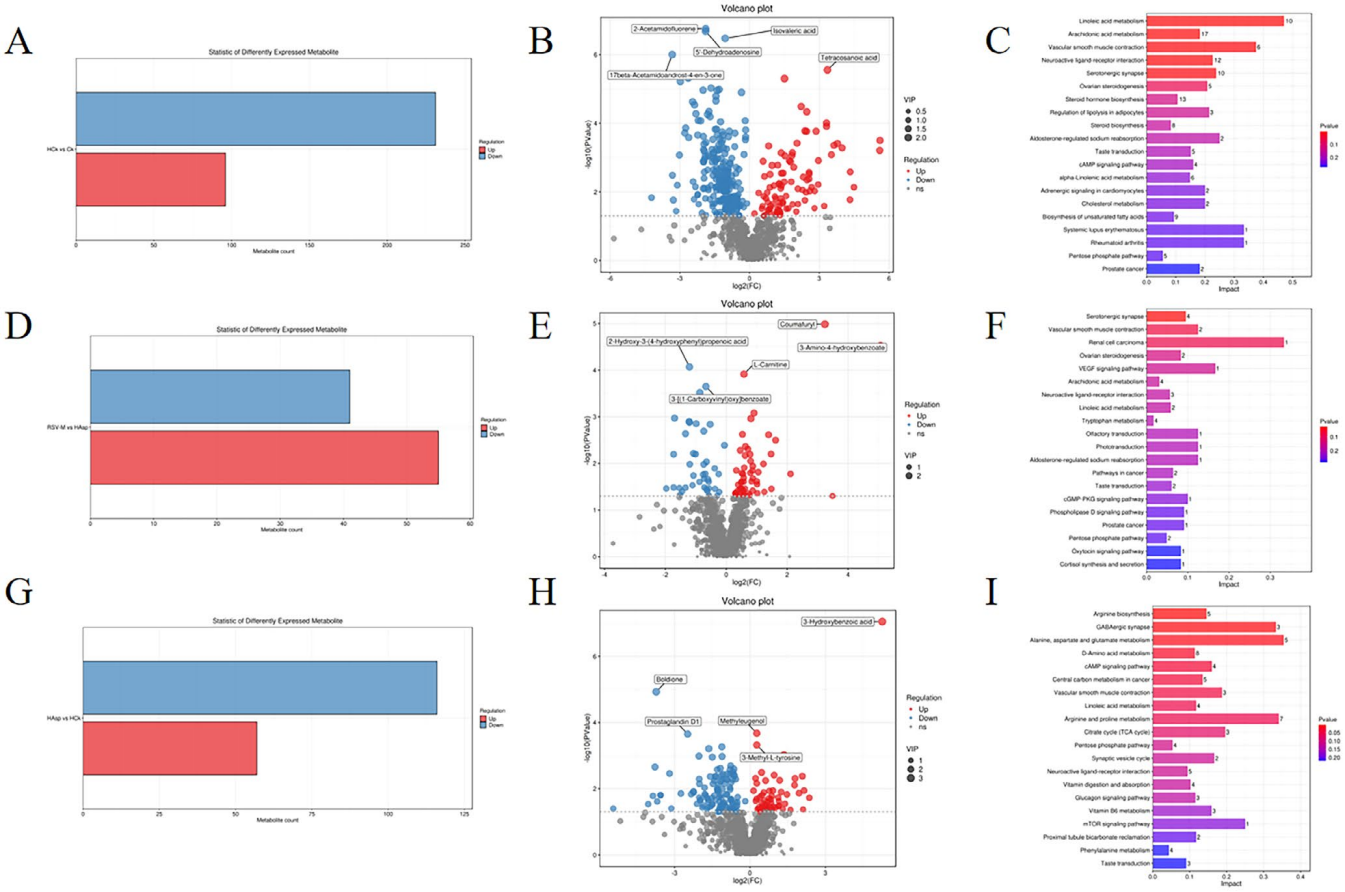
Analysis of rat serum differential metabolites. (A) number of HCk‐Ck, (D) RSVM‐HAsp. (G) HAsp‐HCk differential metabolites. (B) volcano plots of HCk‐Ck. (E) RSVM‐HAsp. (H) HAsp‐HCk differential metabolites. (C) volcano plots of HCk‐Ck. (F) RSVM‐HAsp. (I) HAsp‐HCk differential metabolite KEGG enrichment maps.

A total of 15,449 differential metabolites were detected by LC–MS among the seven groups (Figure 7C,F,I). Compared with the Ck group, 231 metabolites were down‐regulated and 96 metabolites were up‐regulated in the HCk group. The Kyoto Encyclopedia of Genes and Genomes (KEGG) database annotation showed that these differential metabolites were mainly associated with the metabolism of linoleic acid and arachidonic acid as well as vascular smooth muscle contraction. Compared with the HCk group, 116 metabolites were down‐regulated and 57 metabolites were up‐regulated in the HAsp group. These differential metabolites were mainly related to the biosynthesis of arginine, GABAergic synapses, and the metabolism of alanine, aspartate, and glutamate. Compared with the HAsp group, 41 metabolites were down‐regulated and 55 metabolites were up‐regulated in the RSVM group. These differential metabolites were mainly related to serotonergic synapses and vascular smooth muscle contraction.

Further analysis of the differential metabolite results revealed that compared with the Ck group, the HCk group had decreased levels of the metabolites 2‐acetamidofluorene, 5′‐dehydroadenosine, isovaleric acid, and 17 beta‐acetamidoandrost‐4‐en‐3‐one and increased levels of tetracosanoic acid (*p* < 0.05). Compared with the HCk group, the HAsp group had decreased levels of boldione and prostaglandin D1 and increased levels of 3‐hydroxybenzoic acid, methyleugenol, and 3‐methyl‐L‐tyrosine (*p* < 0.05). Compared with the HCk group, the RSVM group had decreased levels of 2‐hydroxy‐3‐(4‐hydroxyphenyl)propenoic acid and 3‐[(1‐carboxyvinyl)oxy]benzoate and increased levels of coumafuryl, 3‐amino‐4‐hydroxybenzoate, and L‐carnitine (*p* < 0.05). These results suggest that RSV can alter the metabolite content in rats with aspirin‐induced intestinal injury under plateau hypoxia (Figure 7B,E,H).

### 3.6 Correlation Analysis

**FIGURE 8:**
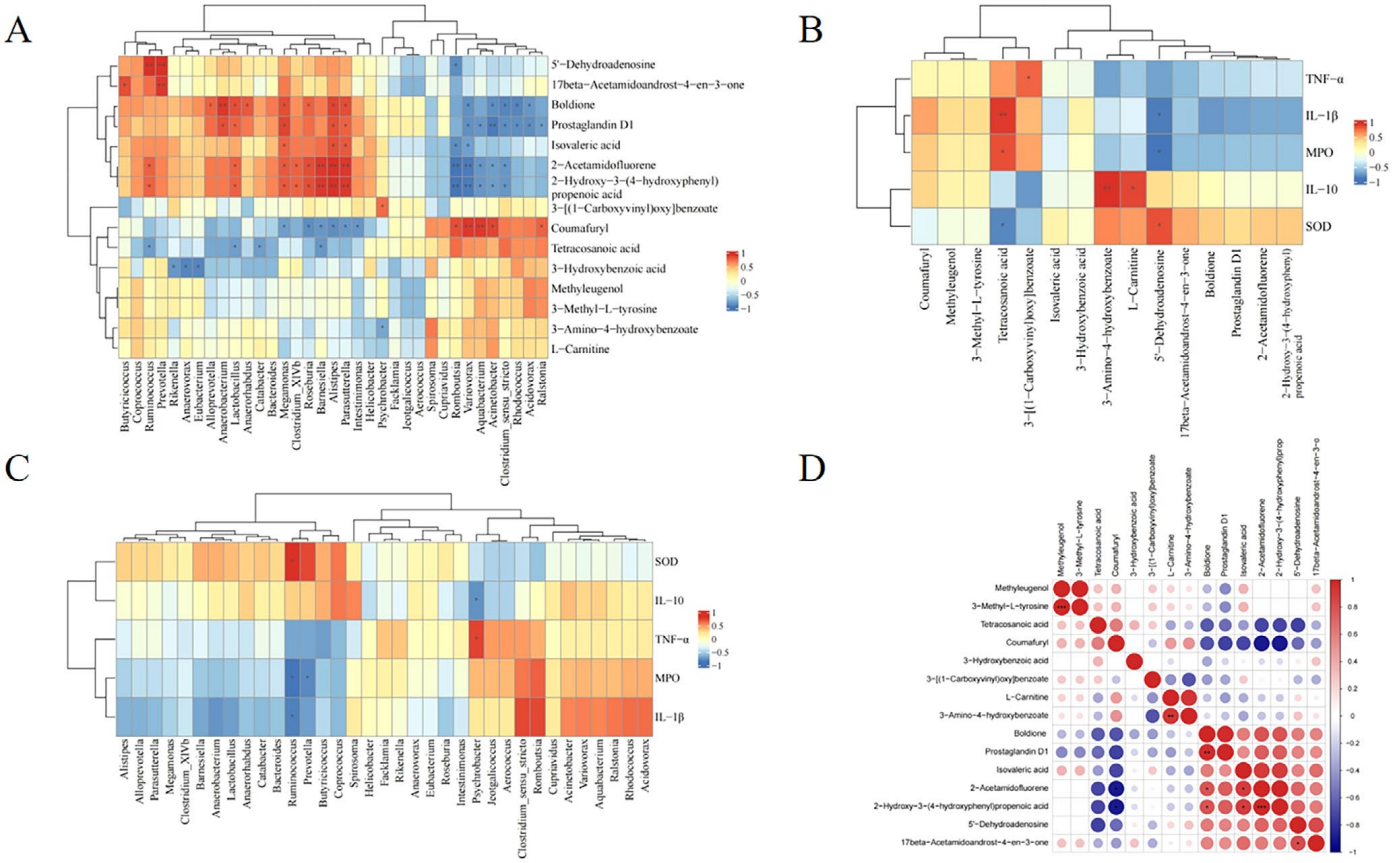
Correlation analysis. (A) Correlation between genus level differential flora and differential metabolites. (B) Correlation between inflammation‐related index and differential metabolites. (C) Correlation between inflammation‐related index and differential flora. (D) Correlation between different metabolites in serum. Positive correlations in red and negative correlations in blue. *p < 0.05.

In addition, we conducted correlation analysis to explore the relationship among intestinal gut inflammation indices (IL‐10, IL‐1β, and TNF‐α), intestinal flora, and differential metabolites (Figure 8). The study results indicated a negative correlation between the abundance of the intestinal flora *Ruminococcus* and the levels of MPO and IL‐1β as well as a positive correlation with SOD content (*p* < 0.05) (Figure 8C). The abundance of *Prevotella* exhibited a negative correlation with the MPO content (*p* < 0.05). *Psychrobacter* abundance demonstrated a negative correlation with IL‐10 expression and a positive correlation with TNF‐α expression (*p* < 0.05). IL‐10 expression exhibited a positive correlation with the content of metabolites 3‐amino‐4‐hydroxybenzoate and L‐carnitine (*p* < 0.05). SOD expression showed a positive correlation with the content of 5′‐dehydroadenosine and a negative correlation with the content of tetracosanoic acid (*p* < 0.05). Both MPO and IL‐1β exhibited a negative correlation with the content of 5′‐dehydroadenosine and a positive correlation with the content of tetracosanoic acid (*p* < 0.05). *Psychrobacter* abundance demonstrated a positive correlation with the content of 3‐[(1‐carboxyvinyl)oxy]benzoate (*p* < 0.05) (Figure 8B). The content of 3‐[(1‐carboxyvinyl)oxy]benzoate exhibited a positive correlation with L‐carnitine content (*p* < 0.05) (Figure 8D).

## 4 Discussion

The plateau hypoxic environment is a primary habitat for human beings, and it can induce various adverse reactions, including headache, dizziness, fatigue, and gastrointestinal symptoms (Richalet, Hermand, et al. 2023). In severe instances, it may lead to intestinal damage and the onset of various intestinal diseases such as irritable bowel syndrome (IBS) and inflammatory bowel disease (IBD), significantly impacting the health and work efficiency of individuals residing in plateau regions (Aragón‐Vela et al. 2020). The administration of NSAIDs under plateau hypoxia may exacerbate intestinal damage significantly. Hence, the focus of this study is on the safety of NSAID medication under plateau hypoxia. The primary research objective of this study is to conduct an in depth exploration of the optimal dosage of drug administration, with the aim of ensuring safety and effectively preventing intestinal damage in the hypoxic environment of the plateau. In this investigation, we selected RSV as a “modifier” to mitigate the intestinal damage induced by NSAIDs in the plateau hypoxic environment. Resveratrol, a polyphenolic compound, possesses anti‐inflammatory, anticancer, and immunomodulatory effects, thereby preventing and treating various intestinal diseases (Wang, Shen, et al. 2023). The objective of this study was to examine the intervention mechanism of RSV in mitigating intestinal injury induced by the administration of NSAIDs under plateau hypoxic conditions. Additionally, we explored its modulatory effects on intestinal microorganisms and serum metabolites in this environment. The findings of this study can offer valuable guidance for preventing intestinal diseases resulting from NSAID use in plateau hypoxia.

In this study, the rat intestinal injury model induced by NSAIDs was mimicked through aspirin gavage. The rats were placed in a hyperbaric hypoxic chamber (Simulated altitude of 5500 m), establishing a rat model of intestinal injury caused by the dual stresses of plateau hypoxia and NSAIDs. Aspirin was found to cause intestinal damage as indicated by H&E staining, suggesting that the induction of intestinal damage caused by NSAIDs was successful. Moreover, the degree of intestinal injury in rats was aggravated under the hypoxic environment of the plateau. In addition, we evaluated the expression levels of pro‐inflammatory cytokines IL‐1β and TNF‐α, alongside the anti‐inflammatory factor IL‐10, across various treatment groups. We observed an elevation in IL‐1β and TNF‐α expression levels, accompanied by a decrease in IL‐10, in the HCk group compared to the Ck group. This implies that the hypoxic plateau environment induces intestinal inflammation, aligning with prior research findings (Pena et al. 2024). In comparison to the HCk group, the HAsp group exhibited further elevation in IL‐1β and TNF‐α expression levels, coupled with a greater reduction in IL‐10 expression. Our findings indicate that administering aspirin in a plateau hypoxic environment exacerbates intestinal inflammation. In contrast to the HAsp group, the RSV intervention group exhibited decreased expression of pro‐inflammatory factors and increased expression of anti‐inflammatory factors. This implies that RSV can effectively mitigate aspirin‐induced intestinal inflammation under plateau hypoxia, potentially attributable to the anti‐inflammatory and antioxidant properties of RSV (Gostimirovic et al. 2023).

Oxidative stress dysregulation is a primary indicator used to assess intestinal damage (Sun et al. 2024). In a state of oxidative stress, there is a significant increase in the production of free radicals, surpassing the body's capacity to scavenge them (Pan et al. 2021). We assessed the levels of myeloperoxidase (MPO) and superoxide dismutase (SOD), which serve as indicators of oxidative stress, across various treatment groups. MPO serves as an objective indicator of centrocyte recruitment, and evaluating MPO activity provides a reliable method for assessing centrocyte infiltration in jejunal tissues (Prakash et al. 2023). The primary function of SOD is to preserve cellular homeostasis and health by reducing intracellular levels of superoxide radicals (Miao and St Clair 2009; Carillon et al. 2013). Studies have demonstrated the potential of RSV to treat various diseases by modulating oxidative stress levels (Rashid et al. 2023; Barreiro‐Sisto et al. 2024). Our study findings indicate that both exposure to a plateau hypoxic environment and aspirin treatment can induce alterations in oxidative stress levels. In the HCk and HAsp groups, the expression of SOD was down‐regulated, while the expression of MPO was up‐regulated compared to the Ck group. Conversely, following RSV intervention, the expression of SOD was up‐regulated, while the expression of MPO was down‐regulated. The results suggest that RSV reversed the down‐regulation of SOD and the up‐regulation of MPO induced by aspirin in the plateau hypoxic environment. Among these groups, the RSVM group (50 mg/kg) exhibited a more pronounced protective effect against oxidative stress‐induced injury in rats with intestinal injury.

Gut microorganisms are the main barrier to maintain intestinal immune homeostasis and prevent the invasion of pathogens (Marchesi et al. 2016). The plateau region is one of the main environments inhabited by human beings. Earlier studies have found that highland hypoxia causes intestinal flora dysbiosis in rats and that NSAIDs also cause changes in the composition of intestinal flora (Qi et al. 2023; Wang et al. 2024). Aspirin, a typical non‐steroidal anti‐inflammatory drug, affects the composition of intestinal flora and causes intestinal mucosal damage when taken for a long period of time. Therefore, by studying the changes in intestinal flora caused by aspirin, it may be helpful to identify appropriate interventions to prevent intestinal damage caused by aspirin. In the present study, the structure of the intestinal microbial community was analyzed by 16SrRNA sequencing. The dominant flora in seven groups of rats were *Bacteroidetes* (CK), *Alloprevotella* (HCk), *Ruminococcus2* (HAsp), *Ruminococcus* (PAsp), *Peptostreptococcaceae* (RSV‐H), *Bilophila* (RSV‐L), *Clostridiales* (RSV‐M). At the genus level, we found that aspirin treatment significantly reduced *Facklamia*, *Jeotgalicoccus*, *Roseburia*, *Psychrobacter*, and *Alloprevotella* (*p* < 0.05). In the RSVM group compared to the HAsp group, Clostridium_sensu_strictoand Spirosoma were significantly higher (*p* < 0.05). It has been shown that RSV can significantly increase the abundance of *Clostridium_sensus_stricto_1* and maintain the integrity of the intestinal barrier by producing short‐chain fatty acids (SCFAs) that promote intestinal barrier health (Ma et al. 2022; Wu et al. 2023). In the present study, a significant increase in the abundance of *Clostridium_sensus_stricto* was found in the RSVM group. These results suggest that RSV can regulate the composition of the intestinal flora and ameliorate intestinal inflammation, with the RSVM group being the most effectively treated.

Changes in intestinal flora induce alterations in metabolites. We utilized LC–MS for analyzing the variations in fecal metabolites among rats subjected to distinct treatment regimens. Treatment in the RSVM group resulted in decreased levels of 2‐hydroxy‐3‐(4‐hydroxyphenyl)propenoic acid and 3‐[(1‐carboxyvinyl)oxy]benzoate, along with elevated levels of coumafuryl, 3‐amino‐4‐hydroxybenzoate, and L‐carnitine. Correlation analysis revealed a positive association between 3‐amino‐4‐hydroxybenzoate, L‐carnitine, and IL‐10 expression. It has been shown that 3‐amino‐4‐hydroxybenzoate has antimicrobial and antitumor effects (Ye et al. 2023), while carnitine can transfer long‐chain fatty acids to the mitochondria for β‐oxidation, which induces energy metabolism, among other effects (Hailemariam et al. 2023). The present study suggests that RSV can modulate metabolite levels and reduce intestinal inflammation, thereby alleviating aspirin‐induced intestinal damage.

In summary, RSV can reduce intestinal inflammation by decreasing the expression of TNF–α and IL—1β. Meanwhile, RSV can regulate the expression levels of SOD and MPO in blood. Given the close relationship between blood and tissues, this regulation may indirectly reflect its impact on the oxidative stress status of jejunal tissues, potentially alleviating the damage to jejunal tissues to a certain extent. Additionally, it can reduce intestinal inflammation and alleviate intestinal injury by regulating the relative abundance of intestinal flora and metabolite content. Hence, RSV can alleviate the intestinal injury induced by prolonged administration of NSAIDs like aspirin in populations residing in plateau hypoxic areas. Nevertheless, this study has some limitations; we did not identify the specific mechanism underlying aspirin‐induced intestinal injury. We merely observed that RSV could alleviate such injury. Hence, it is imperative to conduct comprehensive investigations into the specific mechanisms through which aspirin and other NSAIDs induce intestinal injury. Furthermore, determining whether the administration of such drugs under special circumstances exacerbates or triggers their original side effects is of paramount importance.

## 5 Conclusions

In conclusion, RSV can reduce intestinal inflammation by decreasing the expression of TNF‐α and IL‐1β, and it can also ameliorate jejunal tissue damage by regulating oxidative stress through modulating the expression of SOD and MPO. In addition, RSV can also contribute to the maintenance of intestinal health by regulating intestinal microbes and metabolites. The above results suggest that RSV can be used as a dietary supplement to alleviate aspirin‐induced intestinal injury in rats under plateau hypoxia.

## Author Contributions

**ShengLong Xue:** conceptualization (equal), data curation (equal), formal analysis (equal), investigation (equal), methodology (equal), software (equal), visualization (equal), writing – original draft (equal), writing – review and editing (equal). **Tian Shi:** data curation (equal), resources (equal), supervision (equal), visualization (equal). **Weidong Liu:** formal analysis (equal), investigation (equal), resources (equal). **Yan Feng:** data curation (equal), funding acquisition (equal), project administration (equal), resources (equal), supervision (equal), writing – review and editing (equal). **Ailifeire Tuerxuntayi:** conceptualization (equal), methodology (equal). **Na Li:** supervision (equal). **Feng Gao:** conceptualization (equal), funding acquisition (equal), investigation (equal), project administration (equal), resources (equal), supervision (equal), visualization (equal), writing – review and editing (equal).

## Ethics Statement

This study was approved by the ethics Committee of Xinjiang Uygur Autonomous Region People's Hospital (Xinjiang, China) (protocol n. KY20230209135; approval date: February 2023).

## Conflicts of Interest

The authors declare no conflicts of interest.

## Supporting information


Figures S1–S2.

Table S1.

## Data Availability

Data Availability StatementThe data that support the findings of this study are available from the corresponding author upon reasonable request.
