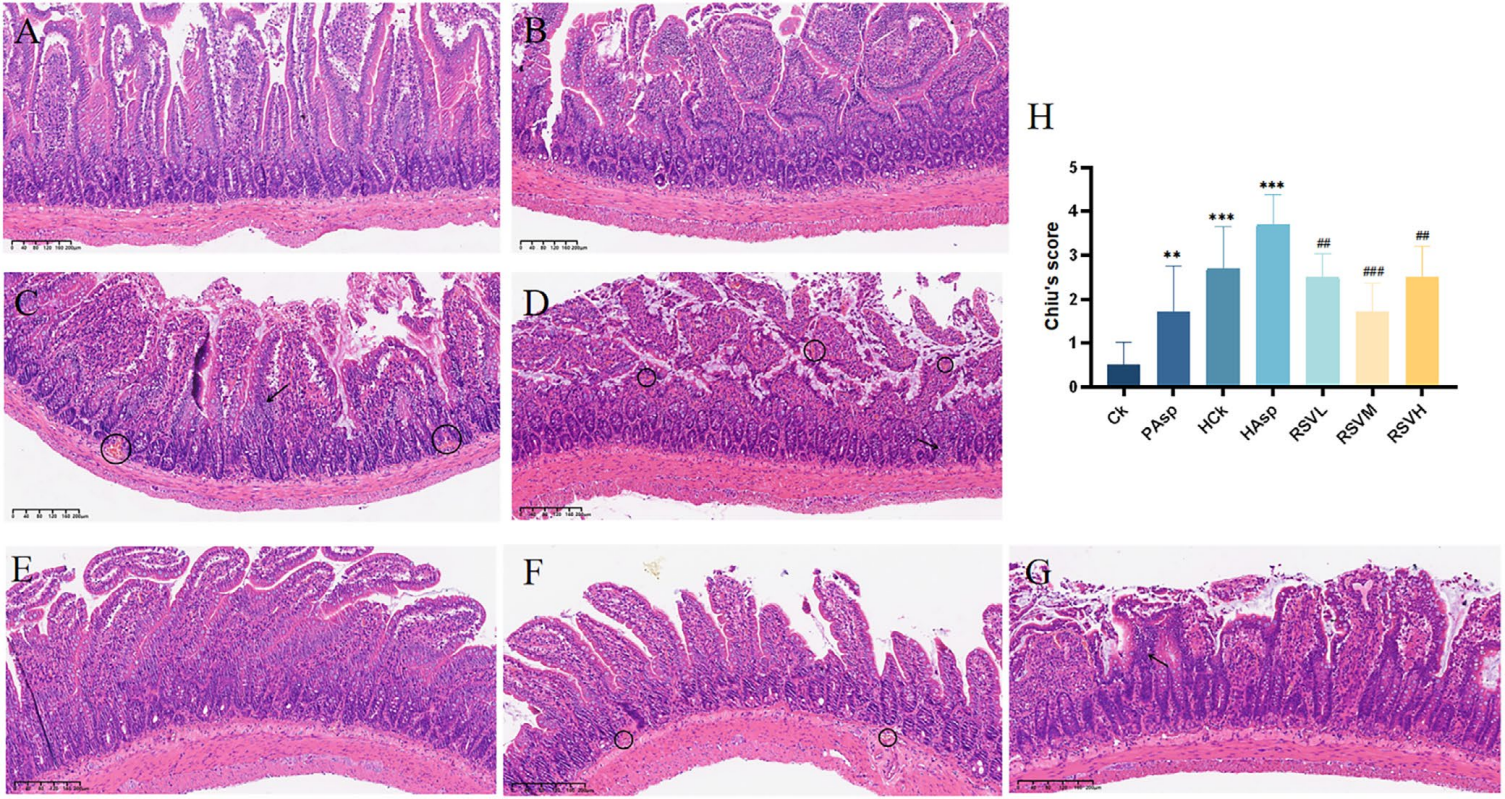# Correction to “Effects of Resveratrol on Intestinal Flora and Metabolism in Rats With Non‐Steroidal Anti‐Inflammatory Drug‐Induced Intestinal Injury Under Plateau Hypoxia Environment”

**DOI:** 10.1002/fsn3.70417

**Published:** 2025-06-11

Xue, S., T. Shi, W. Liu, Y. Feng, A. Tuerxuntayi, N. Li, and F. Gao. 2025. “Effects of Resveratrol on Intestinal Flora and Metabolism in Rats With Non‐Steroidal Anti‐Inflammatory Drug‐Induced Intestinal Injury Under Plateau Hypoxia Environment.” *Food Science & Nutrition* 13: e70228.

This correction is necessary because the images corresponding to Figure 2E and Figure 2F in the article were reversed. In fact, Figure 2E in the original article should represent the medium dose rescue treatment group (RSVM), and Figure 2F should represent the low dose rescue treatment group (RSVL). Therefore, we propose a revision to swap Figure 2E and Figure 2F after the revision. Except for the error in the picture, there are no other errors in the content, which do not affect the conclusion and results of the article.

We apologize for this error.

After modification: Figure 2 after correction: