# Supplementary material for: Effects of Resveratrol on Intestinal Flora and Metabolism in Rats With Non‐Steroidal Anti‐Inflammatory Drug‐Induced Intestinal Injury Under Plateau Hypoxia Environment
Source: Food Sci Nutr. 2025 May 20;13(5):e70228. doi: 10.1002/fsn3.70228 (PMC12121520; doi:10.1002/fsn3.70228)
Supplement: Supplementary file 1 — Figures S1–S2. [file FSN3-13-e70228-s002.zip › FigS1-S2.docx]

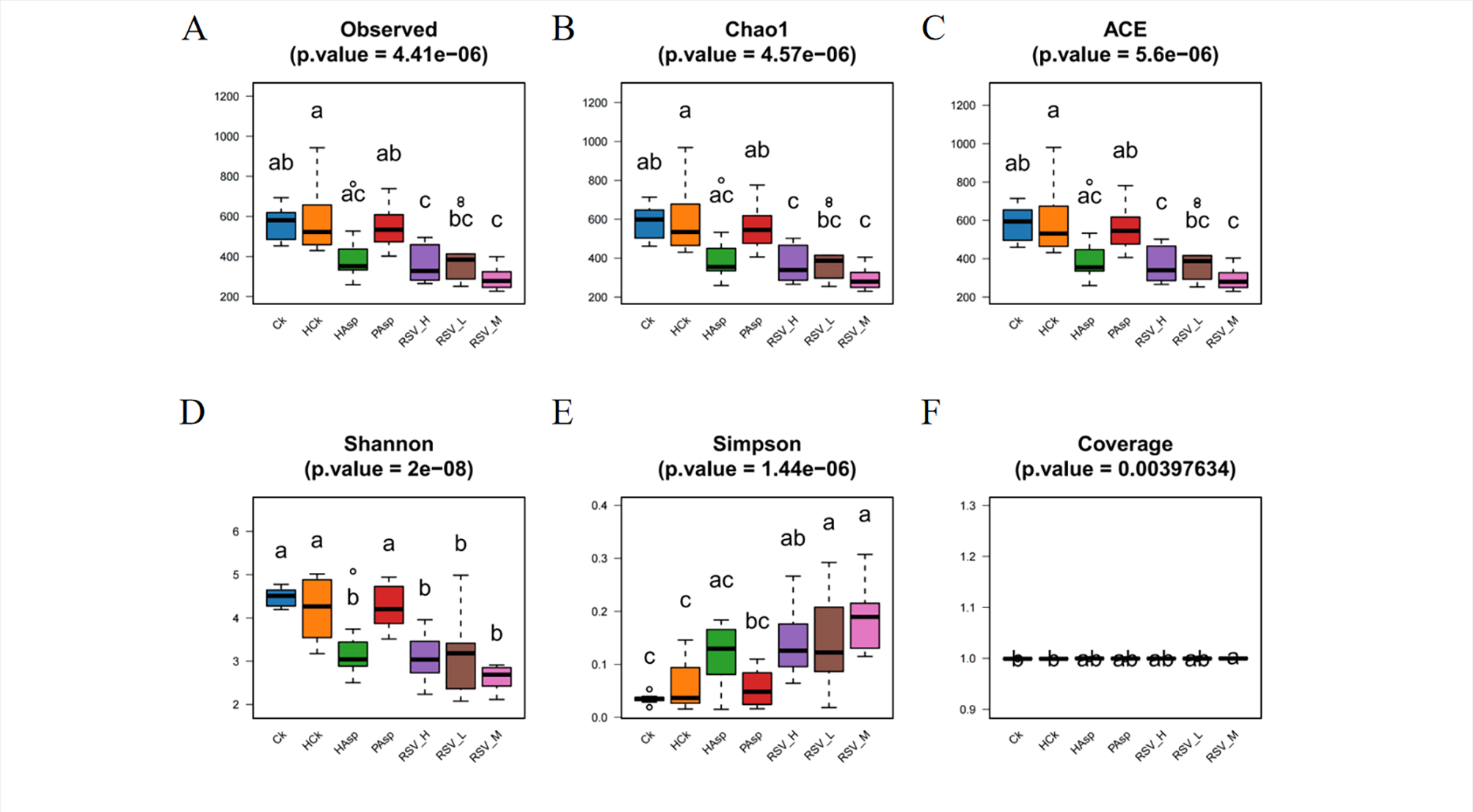


**Supplementary Figure 1. Alpha diversity. (A)** Observed ASVs. **(B)** Chao1 index. **(C)** ACE index. **(D)** Shannon diversity index. **(E)** Simpson index. **(F)** Coverage.


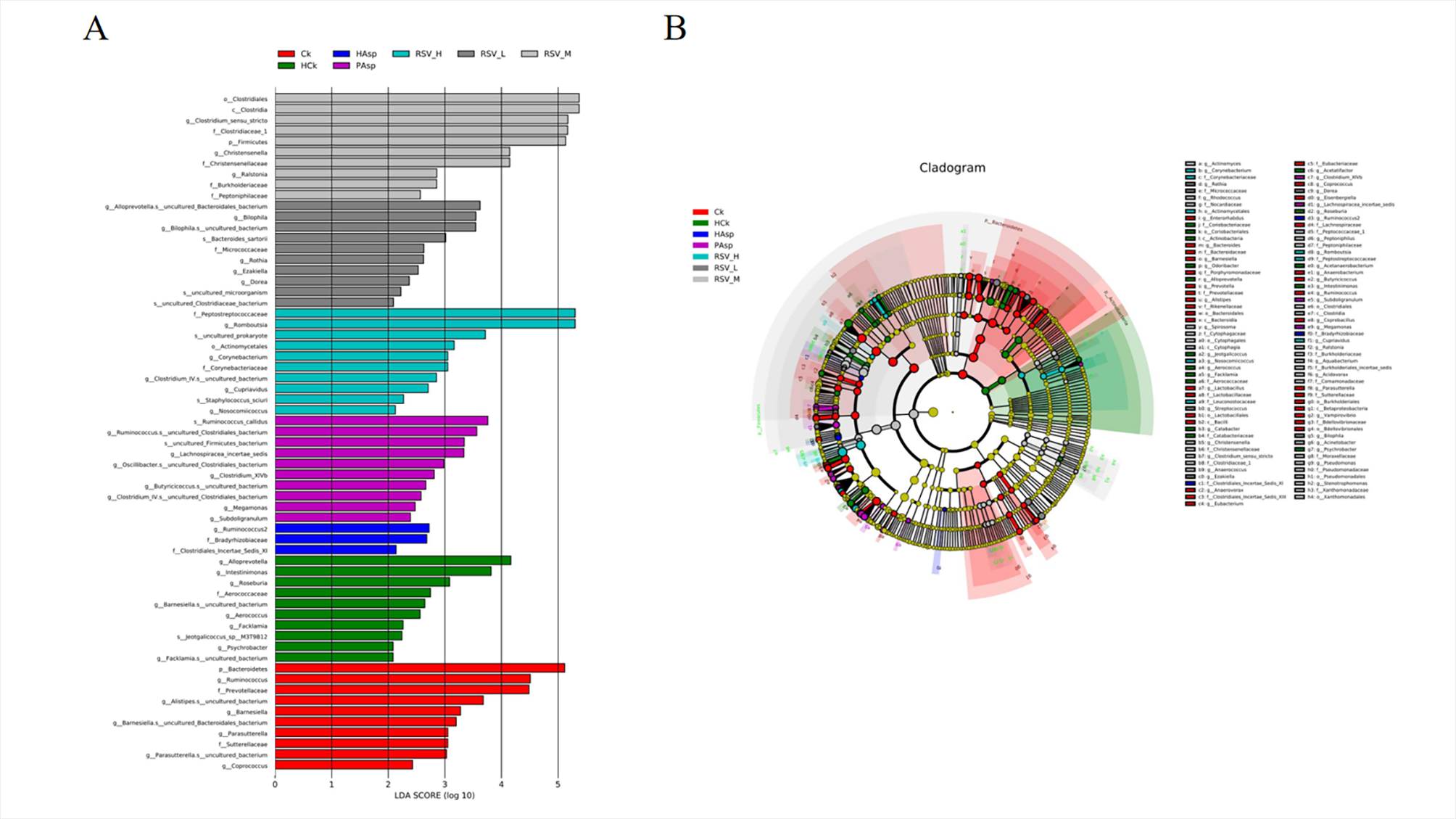


**Supplementary Figure 2. Effect of RSV and Aspirin on LEfSe analysis of rat intestinal flora.** **(A)** LDA plot. **(B)** LEfSe plot.
