# Supplementary figures and images for: Effects of Resveratrol on Intestinal Flora and Metabolism in Rats With Non‐Steroidal Anti‐Inflammatory Drug‐Induced Intestinal Injury Under Plateau Hypoxia Environment
Source: Food Sci Nutr. 2025 May 20;13(5):e70228. doi: 10.1002/fsn3.70228 (PMC12121520; doi:10.1002/fsn3.70228)

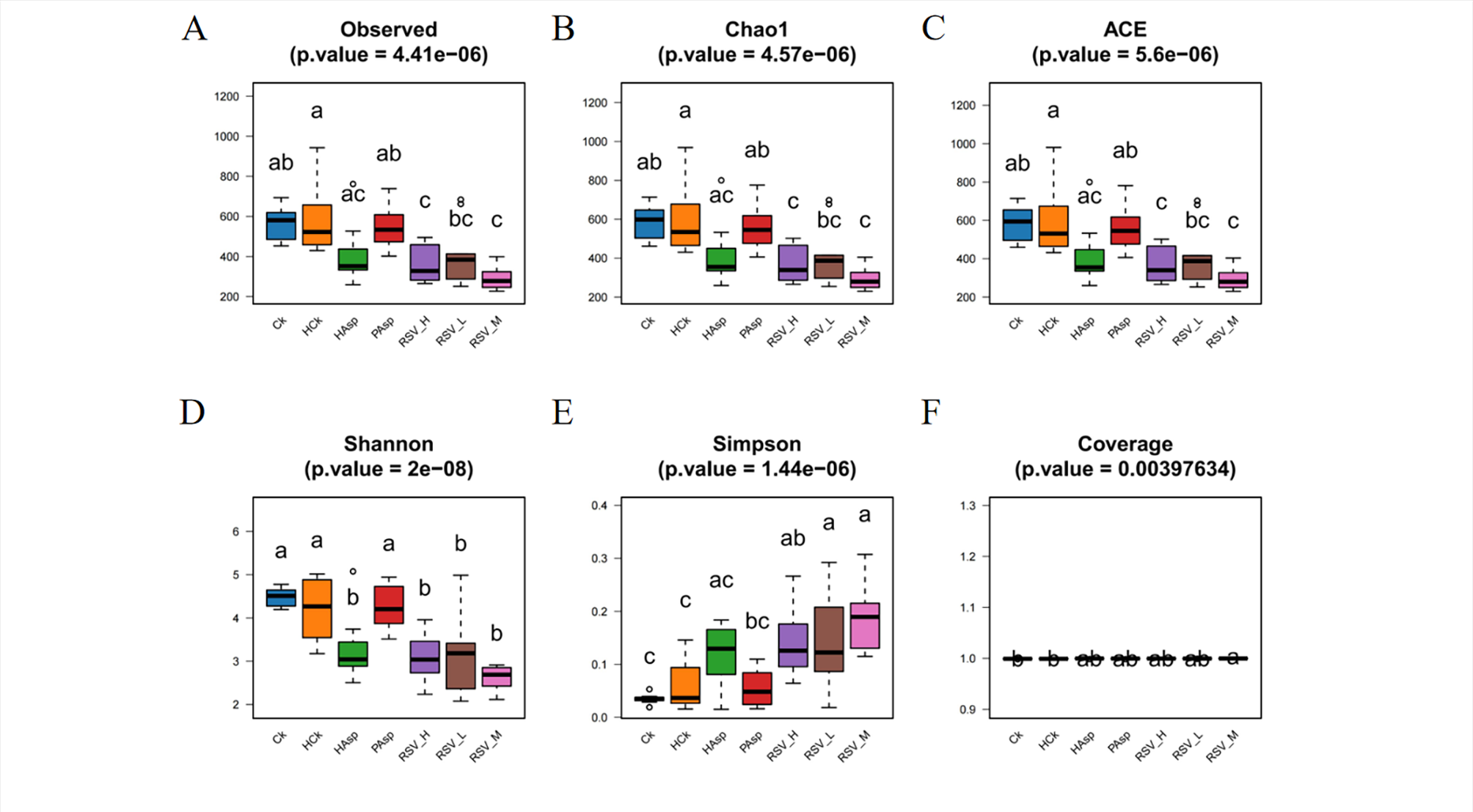

Supplement: Supplementary file 1 — Figures S1–S2. [file FSN3-13-e70228-s002.zip › Supplementary Figure 1 .tif]

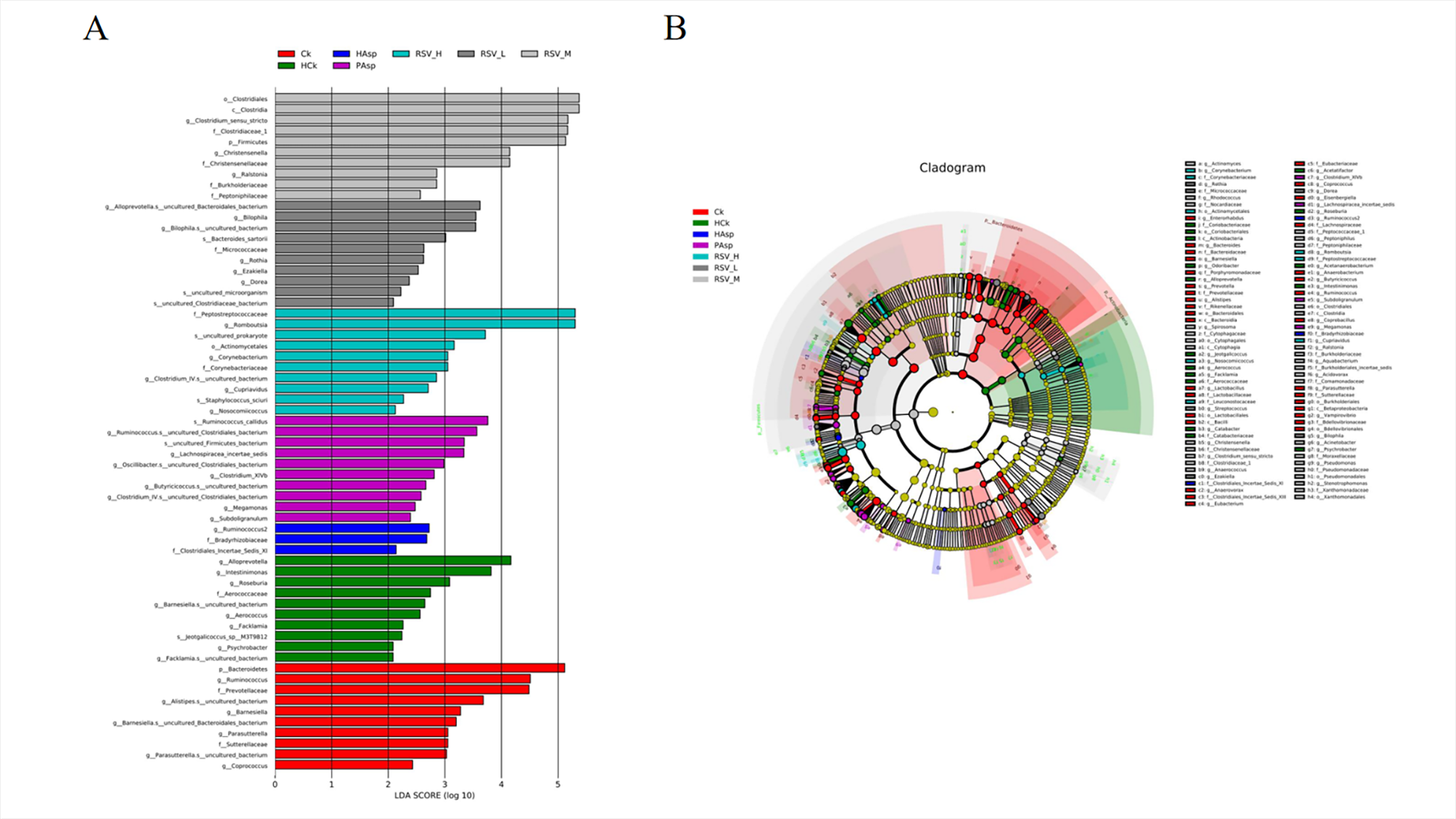

Supplement: Supplementary file 1 — Figures S1–S2. [file FSN3-13-e70228-s002.zip › Supplementary Figure 2.tif]
