# Supplementary material for: Effects of Resveratrol on Intestinal Flora and Metabolism in Rats With Non‐Steroidal Anti‐Inflammatory Drug‐Induced Intestinal Injury Under Plateau Hypoxia Environment
Source: Food Sci Nutr. 2025 May 20;13(5):e70228. doi: 10.1002/fsn3.70228 (PMC12121520; doi:10.1002/fsn3.70228)
Supplement: Supplementary file 2 — Table S1. [file FSN3-13-e70228-s001.docx]

**Supplementary Tables**

**Supplementary Table1** Chiu 's scoring table

| Score | Mucosal condition of the small intestine |
| --- | --- |
| 0 points | Normal intestinal mucosal villi |
| 1 points | Mild subepithelial edema, capillary dilatation and congestion of the apical intestinal mucosa |
| 2 points | Intestinal mucosa with enlarged subepithelial spaces, moderate edema of the lamina propria, and dilated central celiac ducts |
| 3 points | Cellular degeneration and necrosis of the epithelial layer of the intestinal mucosa, marked edema of the lamina propria, and apical detachment of a few villi |
| 4 points | Degeneration, necrosis, detachment of the epithelial cell layer of the intestinal mucosa, exposure of the lamina propria, dilatation and congestion of the capillaries, and detachment of some villi |
| 5 points | Shedding of intestinal mucosal villi, disintegration of lamina propria, bleeding or ulcer formation |
